# Metformin protects against intestinal barrier dysfunction *via* AMPKα1‐dependent inhibition of JNK signalling activation

**DOI:** 10.1111/jcmm.13342

**Published:** 2017-11-17

**Authors:** Jun Deng, Lishan Zeng, Xueying Lai, Jing Li, Le Liu, Qianyun Lin, Ye Chen

**Affiliations:** ^1^ State Key Laboratory of Organ Failure Research Guangdong Provincial Key Laboratory of Gastroenterology Department of Gastroenterology Nanfang Hospital Southern Medical University Guangzhou Guangdong China

**Keywords:** inflammatory bowel disease, intestinal barrier, metformin, tight junction, AMP‐activated protein kinase, C‐Jun N‐terminal kinase

## Abstract

Disruption of the intestinal epithelial barrier, that involves the activation of C‐Jun N‐terminal kinase (JNK), contributes to initiate and accelerate inflammation in inflammatory bowel disease. Metformin has unexpected beneficial effects other than glucose‐lowering effects. Here, we provided evidence that metformin can protect against intestinal barrier dysfunction in colitis. We showed that metformin alleviated dextran sodium sulphate (DSS)‐induced decreases in transepithelial electrical resistance, FITC‐dextran hyperpermeability, loss of the tight junction (TJ) proteins occludin and ZO‐1 and bacterial translocation in Caco‐2 cell monolayers or in colitis mice models. Metformin also improved TJ proteins expression in ulcerative colitis patients with type 2 diabetes mellitus. We found that metformin ameliorated the induction of colitis and reduced the levels of pro‐inflammatory cytokines IL‐6, TNF‐a and IL‐1β. In addition, metformin suppressed DSS‐induced JNK activation, an effect dependent on AMP‐activated protein kinase α1 (AMPKα1) activation. Consistent with this finding, metformin could not maintain the barrier function of AMPKα1‐silenced cell monolayers after DSS administration. These findings highlight metformin protects against intestinal barrier dysfunction. The potential mechanism may involve in the inhibition of JNK activation *via* an AMPKα1‐dependent signalling pathway.

## Introduction

The gastrointestinal epithelium is a multilayered barrier that functions as a defence system against pathogens and other harmful compounds in the lumen. Increasing evidence indicates that the epithelial barrier dysfunction contributes to the onset and development of various diseases, including inflammatory bowel disease (IBD) 1. IBD, comprises ulcerative colitis (UC) and Crohn's disease (CD), is associated with a chronic and relapsing inflammation condition. One of the most typical feature of IBD is epithelial barrier disruption, which contributes to IBD pathogenesis 1, 2.

The epithelial barrier comprises epithelial cells and intercellular junctional complexes 3, including TJs. The transmembrane proteins of TJs, namely, occludin and claudins, interact with the central plague protein ZO‐1, which associates with the cytoskeleton 4. This junction complex plays a critical role in forming and maintaining the epithelial barrier. Defective epithelial TJs cause paracellular barrier functional disturbances and increase the paracellular permeability of intestinal epithelium. Abnormally high paracellular permeability results in intestinal penetration by luminal bacteria and potentially harmful antigens, which appear to initiate and accelerate inflammation in IBD 1, 5, 6, 7. Therefore, improving intestinal barrier function can alleviate inflammation development or accelerate inflammation resolution 6, 8.

Metformin has been widely used for the treatment of type 2 diabetes mellitus (T2DM). Recently, researchers have uncovered increasing amounts of evidence that metformin has additional beneficial effects beyond facilitating improvements in glucose homoeostasis. For example, studies have reported that metformin plays a role in anti‐ageing and increase lifespan 9, 10. Metformin has also been shown to partially attenuate gut dysbiosis caused by disease 11, 12. Furthermore, many studies have shown that metformin has anti‐inflammatory effects *in vitro* and *in vivo* 13, 14, 15, 16. Specifically, some studies have reported that metformin can attenuate intestinal inflammation in mice with colitis 17, 18.

Intestinal epithelial barrier function and inflammation are closely related, and metformin has anti‐inflammatory property in the gut. However, there is little evidence that whether metformin can protect intestinal epithelial barrier function. In this study, we showed that metformin protects intestinal epithelial barrier function *in vitro* and *in vivo* and elucidated its possible mechanism *via* AMPKα1‐dependent inhibition of JNK signalling pathway.

## Materials and methods

### Cell culture

Caco‐2 cells were obtained from American Type Culture Collection and maintained in Dulbecco's modified Eagle medium (DMEM), supplemented with 100 U/ml penicillin, 100 mg/ml streptomycin and 20% (v/v) foetal bovine serum (FBS). The medium was refreshed every 2–3 days, and cells were passaged using 0.05% trypsin.

### Transepithelial electrical resistance (TEER) measurement

A total of 1 × 10^5^ Caco2 cells were seeded in 24‐well transwell chambers (6.5 mm diameter, 3 μm pore size; Corning, Tewksbury, MA, USA) and cultured for 15 days. The integrity of cellular monolayer was determined by measuring TEER values. When the TEER values were consistently above 300 Ω cm^2^, Caco‐2 cells monolayers were considered to have been matched for the experiment 19; 3% DSS (MP Biochemical, Santa Ana, CA, USA) dissolved in DMEM was added to the upper chamber for 8 hrs after pre‐treatment with metformin, and TEER values was measured.

### Tissue samples

Paraffin‐embedded colonic tissue samples from 22 UC patients with T2DM and 20 normal controls were randomly obtained on endoscopic examination from the Department of Gastroenterology, Nanfang Hospital during 2014–2016. Definitive diagnosis of UC was established by standard endoscopic, histological and clinical criteria. The partial Mayo score was determined previously as described 20. Patients with septic complications, short bowel syndrome or cancer, and pregnant women were excluded. The patients were divided into metformin group and insulin group according to basic treatment of T2DM. The study was carried out in accordance with the institutional ethical guidelines and had been approved by the medical ethics committee of Southern Medical University (number: NFEC‐2014‐035). The sociodemographic and clinical data are summarized in Table S1.

### Animals

Wild‐type male C57BL/6 mice (6–8 weeks, 20–23 g in weight) were housed in specific pathogen‐free conditions. The mice were randomly divided into four groups: a control group, a DSS group, a DSS plus 100 mg/kg/day metformin group and a DSS plus 500 mg/kg/day metformin group. Acute colitis was induced by administrating 3% DSS for 7 days. Metformin were administered *via* gavage for 7 days before colitis induction and then administered in parallel with DSS. The disease activity index (DAI) was calculated as previously described 21. After 7 days, the animals were killed. Colon length was measured, and the colon, livers, spleens, mesenteric lymph nodes (MLNs) and serum samples were collected. Animal handling was approved by the Animal Experimental Ethics Committee of Southern Medical University (number: L2015059).

### Histological analysis

The distal colon was fixed in 4% paraformaldehyde, and then, they were dehydrated with a graded ethanol series, cleared in dimethylbenzene, embedded in paraffin and stained with haematoxylin and eosin (H&E). The degree of intestinal inflammation was assessed according to the modified scoring system devised by Dieleman *et al*. 22. The scores were assessed by two pathologists in double‐blinded manner.

### Quantitative real‐time PCR (qRT‐PCR) analysis

Total RNA from distal colon was isolated using RNAiso Plus (TaKaRa, Dalian, China). First‐strand cDNA was synthesized, and qRT‐PCR was performed with a Roche LightCycler^®^ 480II using SYBR Premix Ex Taq (TaKaRa). The primer sequences are listed in Table S2. The expression level of each mRNA was calculated using the 2^−ΔΔ Ct^ method after the corresponding sample was normalized to the expression level of β‐actin level.

### Enzyme‐linked immunosorbent assay (ELISA) analysis

Serum samples were obtained from the different groups, and protein levels of the inflammatory factors IL‐6, TNF‐α, IL‐1β were determined using commercially available ELISA kits (MultiSciences, Hangzhou, China), according to the manufacturer's protocol.

### Western blot analysis

Total protein was extracted with cold radioimmunoprecipitation lysis buffer, protease inhibitor and phosphatase inhibitor cocktail. Equal amounts of proteins were separated by 8% or 10% SDS‐polyacrylamide gels and then transferred to a PVDF membrane (Bio‐Rad, Marnes‐la‐Coquette, France), which was blocked with 5% skim milk prepared in tris‐buffered saline with Tween (TBST). Then, the membrane was incubated with primary antibodies against GAPDH (ZSGB‐BIO, Beijing, China),ZO‐1 (Invitrogen, Carlsbad, CA, USA), occludin (Protein Tech Group, Inc., Wuhan, China), JNK1/2, p‐JNK1/2, AMPKα, p‐AMPKα (Cell Signaling Technology Inc., Danvers, MA, USA), AMPKα1, AMPKα2 (Abcam, Cambridge, MA, USA) overnight at 4°C. Following washes in TBST, the membrane was subsequently incubated with the appropriate HRP‐conjugated secondary antibodies for 1 hr and then visualized *via* enhanced chemiluminescence detection. Protein expression was quantified by densitometric analysis using ImageJ software (National Institutes of Health, Bethesda, MD, USA).

### Immunohistochemical analysis

The immunohistochemical methods have been described previously 23. Sections were deparaffinized in xylene and rehydrated through a graded ethanol series. Then, they were antigen retrieved, quenched of endogenous peroxidase and incubated with primary antibodies against occludin and ZO‐1 (dilution 1:100) overnight at 4°C. After washing with PBS three times, tissues were incubated with a second antibody (ZSGB‐BIO), developed with the DAB reagent and counterstained with haematoxylin. Negative controls were incubated without antibody. The quantification of TJ proteins was assessed according to the staining intensity and the percentage of the stained epithelial cells. Briefly, the staining intensity was scored as negative (0), weak (1), moderate (2) or strong staining (3). The percentage of positive cells was graded on a scale of 0–4: 0, less than 5%; 5–25% scored 1; 26–50% scored 2; 51–75% scored 3 and more than 75% scored 4. The final score was calculated by multiplying intensity score and percentage score.

### Intestinal permeability *in vivo* and *in vitro*

For the *in vivo* permeability assay, all mice were fasted for 8 hrs and then gavaged with FITC‐dextran (4kD, FD4; Sigma‐Aldrich, St. Louis, MO, USA) at a concentration of 600 mg/kg bodyweight. After 2 hrs, they were anesthetized and their plasma was collected in the dark. FD4 permeability may be partially affected by change in gastrointestinal motility, although the influence of gastrointestinal motility is very limited. For the *in vitro* permeability assay, 1 mg/ml FD4 was added to upper chamber after Caco‐2 cells monolayers being treated. FD4 flux was measured by placing 100 μl solutions from the basolateral chamber after 2 hrs. The fluorescence intensity was measured using a fluorescence spectrophotometer (485 nm excitation and 535 nm emission). FD4 concentration was obtained from standard curves generated by serial FD4 dilution.

### Bacterial translocation in colitis

Total bacterial DNA from MLNs, livers and spleens were extracted using a QIAamp DNA Mini Kit (Qiagen, Hilden, Germany). DNA was amplified using universal 16s rDNA primers (5′‐AGAGTTTGATCATGGCTCAG‐3′, 5′‐ACCGCGACTGCTGCTGGCAC‐3′) for all eubacteria. The amplified product was purified. The plasmid was synthesized by Shanghai Biotechnology Company. To analyse the quantities of bacteria, we used 100 ng DNA to quantitate the bacteria by qRT‐PCR analysis. The copies were quantitated using standard curves constructed with known plasmid DNA concentrations.

### Immunofluorescence staining

Caco‐2 monolayers were fixed in 4% paraformaldehyde. Following permeabilization in 0.1% Triton X‐100, they were blocked in 1% bovine serum albumin. Then, the monolayers were incubated with a primary antibody against ZO‐1 (1:50) and occludin (1:50) overnight at 4°C. The cells were subsequently incubated with the appropriate secondary fluorescence antibody for 1 hr. DAPI was used to stain the cell nuclei. The fluorescence was examined under a confocal laser scanning microscope (Fluoview FV10i; Olympus, Tokyo, Japan).

### Transfection of small interfering RNA (siRNA)

AMPKα1 or AMPKα2 siRNA and negative control (NC) siRNA were obtained from GenePharma (Shanghai, China). The target sequences of siRNA are listed in Table S3. Caco2 cells were transfected with each siRNA (100 nM) using Lipofectamine 3000 Transfection Reagent (Invitrogen) and incubated in Opti‐MEM^®^ I reduced serum medium (Invitrogen) for 8 hrs, according to the manufacturer's instructions. The interfering efficiency of each target gene was determined by Western blot analysis.

### Statistical analysis

All data were representative of at least three independent experiments and were expressed as the mean ± S.E.M. Student's *t*‐test or one‐way analysis of variance (anova) and an appropriate *post hoc* Dunnett's or Tukey's comparison were performed to determine the significance of the differences among the groups. *P* < 0.05 was considered statistically significant.

## Results

### Metformin alleviates DSS‐induced barrier dysfunction in Caco‐2 cell monolayers

**Figure 1:**
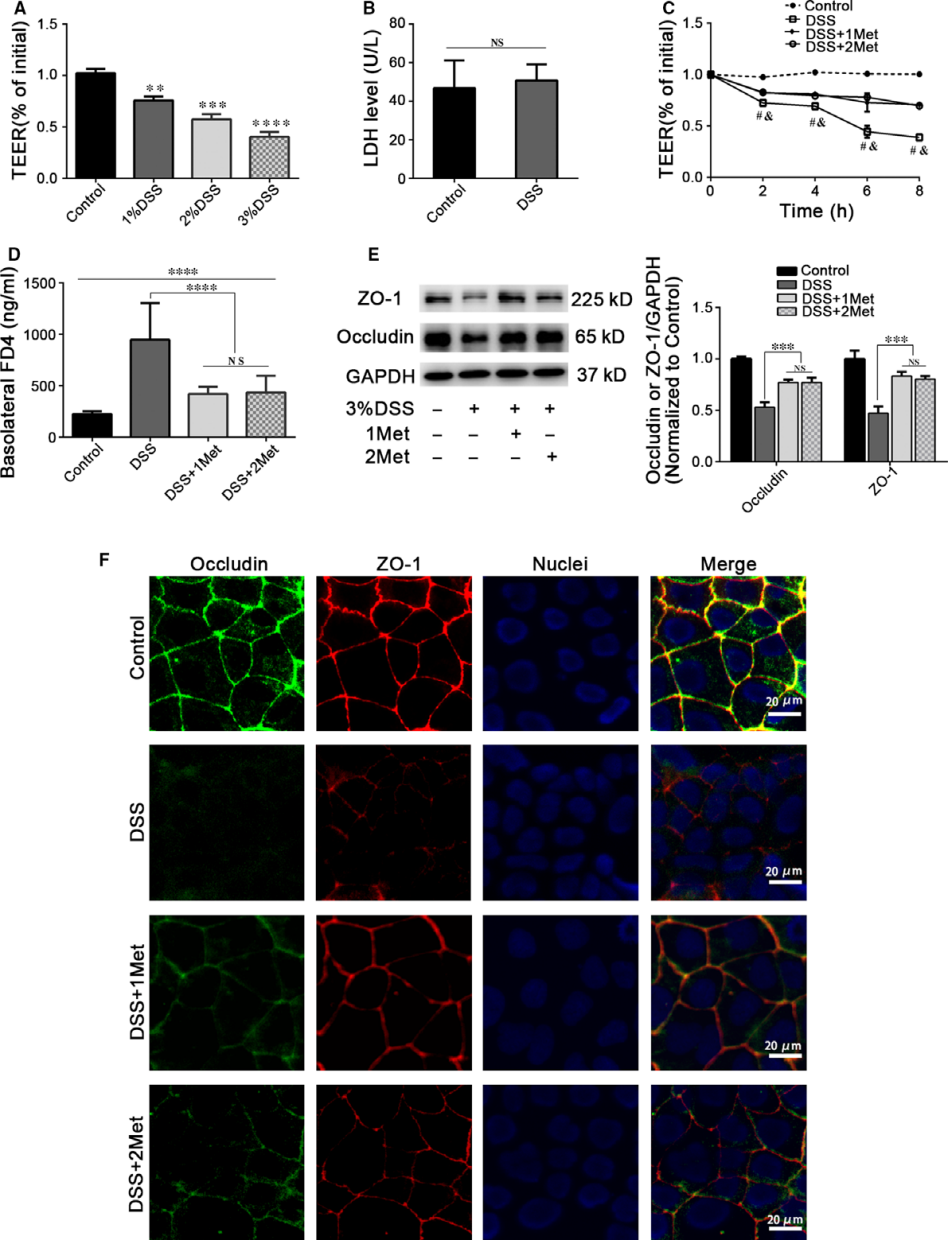
The protective effects of metformin on DSS‐induced barrier dysfunction *in vitro*. 1 × 10^5^ Caco‐2 cells were seeded in 24‐well transwell inserts and cultured for 15 days. (**A**) 1%~3% DSS treated for 8 hrs and TEER value was measured. (**B**) Cell viability was evaluated by measuring lactate dehydrogenase (LDH) activity in medium after 3% DSS treatment for 8 hrs. (**C**) 1 mM / 2 mM metformin pre‐treatment for 2 hrs, followed by coprocessing with 3% DSS for 8 hrs. TEER value was measured. (**D**) After 8 hrs, 1 mg/ml FD4 was added to upper chamber and incubated for 2 hrs. The FD4 flux was measured. (**E**) Expression levels of TJ proteins ZO‐1 and occludin were determined by Western blotting. Band densities were evaluated by Image J software. (**F**) Expression of ZO‐1 (red) and occludin (green), as determined by immunofluorescence. The cell nuclei were stained with DAPI (blue). The images were taken by confocal microscopy (scale bars, 20 μm). Values represent mean ± S.E.M. (*n* = 4). 1Met: 1 mM metformin; 2Met: 2 mM metformin; NS, not significant. ^#^ *P* < 0.001, DSS *versus* DSS+1Met, ^&^ *P* < 0.001, DSS *versus* DSS+2Met. **P < 0.01; ***P < 0.001; ****P < 0.0001.

A ‘leaky’ barrier is attributable to TJ disruption, which alters TEER and paracellular permeability 1, 24. To confirm whether metformin can protect barrier function, we first used 1%~3% DSS to treat Caco‐2 cell monolayers for 8 hrs and observed dose‐dependent decrease in TEER (Fig. 1A). 3% DSS did not induce significant changes in cell viability (Fig. 1B), suggesting the barrier dysfunction was not caused by cell death. After DSS treatment,we noted a significant time‐dependent decrease in TEER. Treatment with metformin (1 mM / 2 mM) could significantly alleviate the TEER decreases, but there is no significant difference between the two concentrations (Fig. 1C). The DSS‐induced transepithelial high permeability of FD4 was significantly reduced by metformin treatment (Fig. 1D). The levels of TJ proteins ZO‐1 and occludin was also significantly decreased after DSS exposure, and they were reversed when combined with metformin treatment (Fig. 1E). Immunofluorescence staining showed that DSS treatment led to both ZO‐1 and occludin depletion and discontinuity. Metformin could attenuate these changes (Fig. 1F).

### Metformin ameliorates the severity of DSS‐induced acute colitis in mice

**Figure 2:**
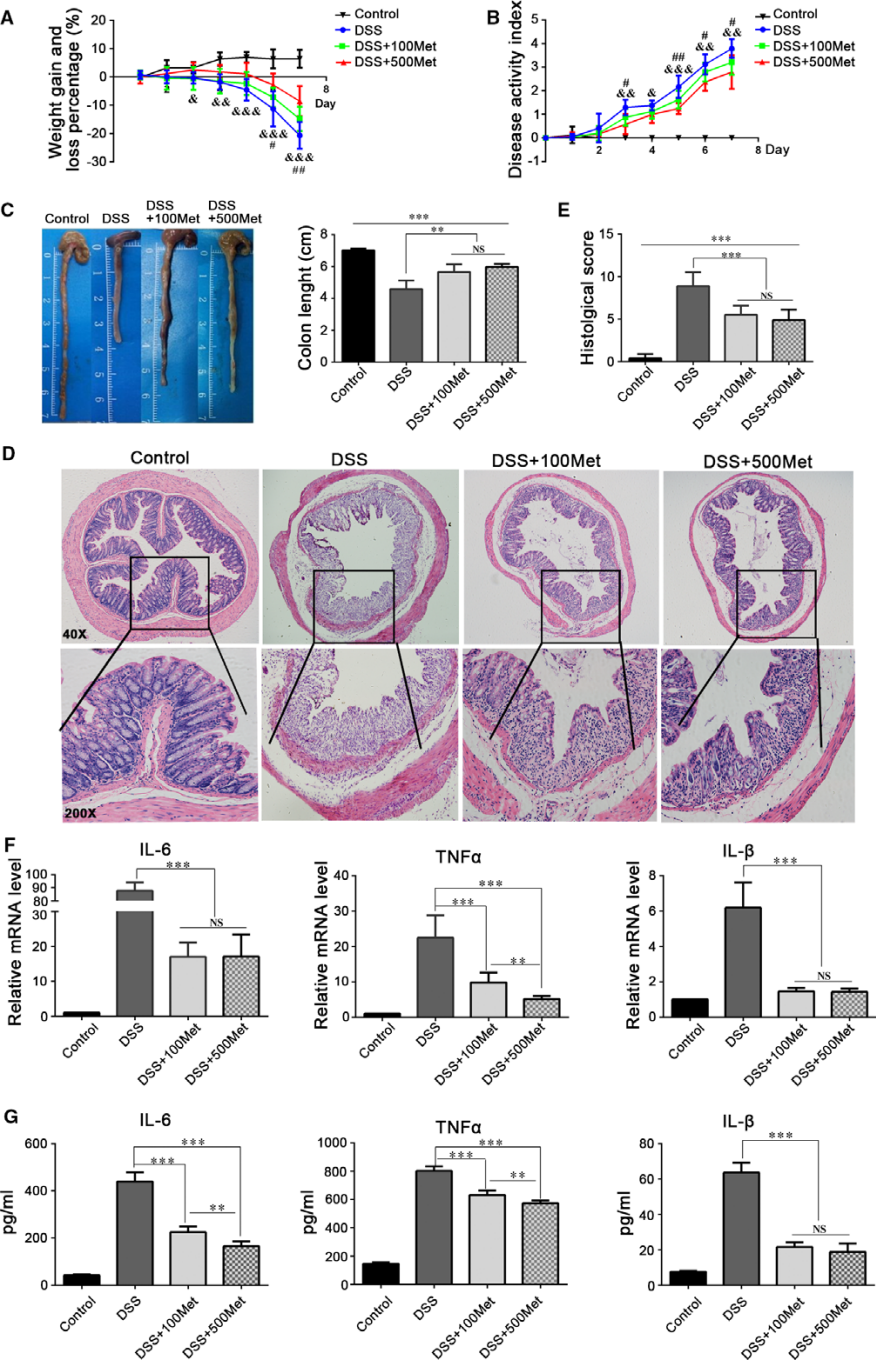
Metformin alleviates the severity of DSS‐induced colitis in mice. C57BL/6 mice were given 3% DSS to induce acute colitis. The metformin groups were administered one of the following two different doses: 100 mg/kg/d and 500 mg/kg/d. (**A**) Bodyweight. (**B**) DAI scores. (**C**) Colon length. (**D**) Histological analysis, H&E staining of the proximal colon; overview: 40 × ; magnification: 200 × . (**E**) Quantitation of histological scores. (**F**) The mRNA levels of IL‐6, TNF‐α and IL‐1β in colon. (**G**) The levels of IL‐6, TNF‐α and IL‐1β in serum, as demonstrated by ELISA. 100 Met: 100 mg/kg/d metformin, 500 Met: 500 mg/kg/d metformin. ^#^ *P* < 0.05; ^##^ *P* < 0.01, DSS+100Met *versus* DSS; ^&^ *P* < 0.05; ^&&^ *P* < 0.01; ^&&&^ *P* < 0.001, DSS+500Met *versus* DSS, **P < 0.01, ***P < 0.001.

To determine whether metformin has beneficial effect in colitis, we treated mice with 3% DSS to induce acute colitis. Mice treated with metformin simultaneously experienced significantly less bodyweight loss than those treated with DSS only. Mice treated with 500 mg/kg/day metformin had less bodyweight loss than those with 100 mg/kg/day. (Fig. 2A). Consistent with these findings, metformin could significantly reduce the DAIs (Fig. 2B) as well as the colon length shortening of DSS‐induced mice (Fig. 2C). Histological examination showed less intestinal mucosal morphological damage, less inflammatory cells infiltration and better histological scores in metformin‐treated mice (Fig. 2D and E). Moreover, we assessed the mRNA and protein levels of pro‐inflammatory cytokines IL‐6, TNF‐a and IL‐1β in colon and in serum, and found that metformin could significantly inhibit the expression of those cytokines (Fig. 2F and G).

### Metformin improves intestinal permeability and promotes TJ expression in colitis

**Figure 3:**
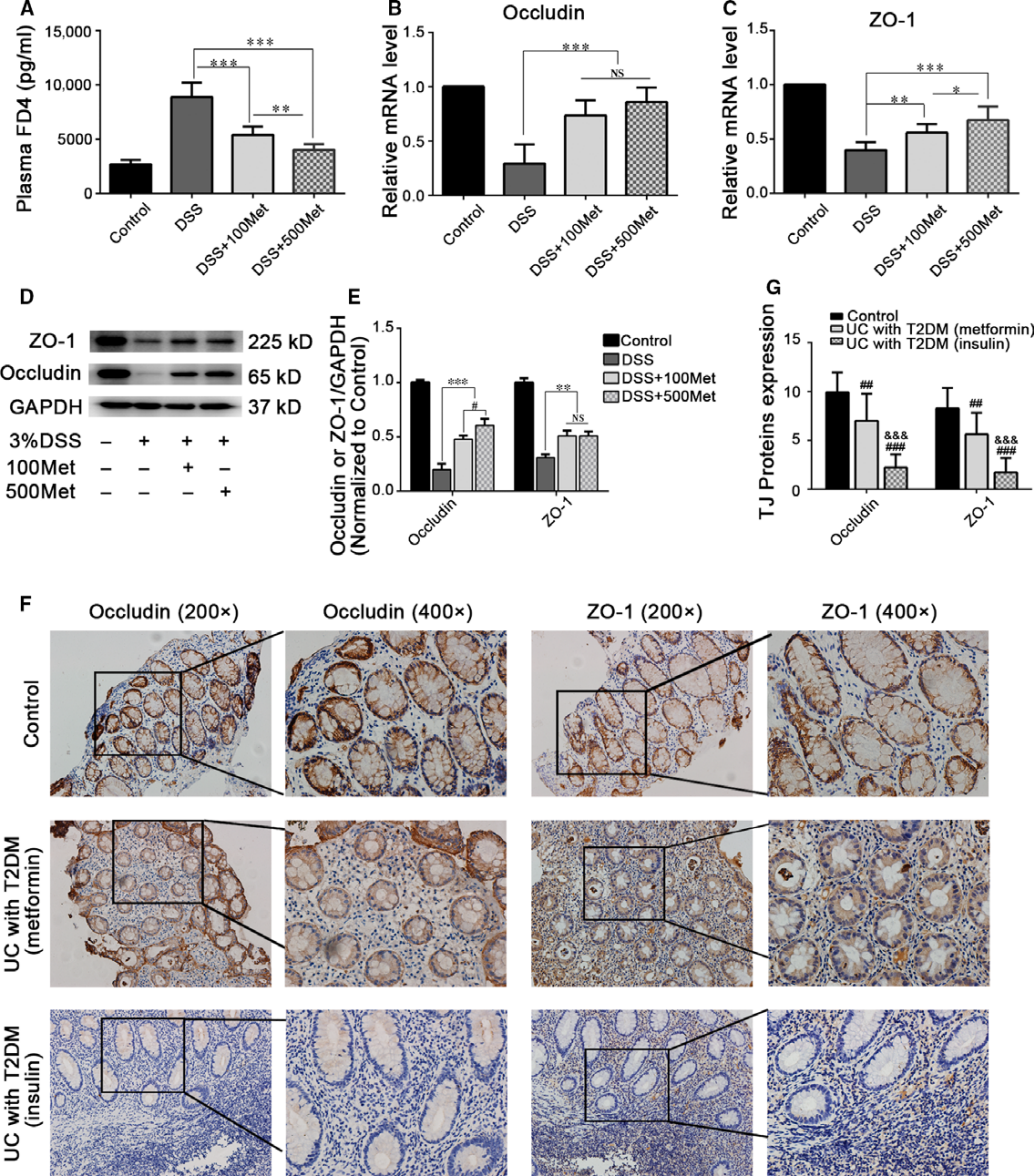
The protective effects of metformin on intestinal barrier function in colitis. (**A**) All animals were fasted for 8 hrs and then gavaged with FD4 at a concentration of 600 mg/kg bodyweight. Plasma samples were collected 2 hrs later, and the fluorescence intensity was measured. (**B–C**) The mRNA expression levels of TJ proteins occludin and ZO‐1 in colonic mucosa. (**D–E**) Western blotting detected the levels of TJ proteins in colonic mucosa, and band densitometric analysis was performed. (**F–G**) Representative immunostaining and quantitative analysis of occludin and ZO‐1 in intestinal mucosa of UC patients with T2DM, overview: 200×; magnification: 400×. ^##^ *P* < 0.01; ^###^ *P* < 0.001, *versus* control; ^&&&^ *P* < 0.001, *versus* UC with T2DM (metformin); *P < 0.05, **P < 0.01; ***P < 0.001.

Intestinal barrier dysfunction leads to intestinal inflammation 25. We speculated that the anti‐inflammation effect of metformin is probably facilitated by maintaining intestinal barrier function. To confirm this hypothesis, we first assessed intestinal permeability. Metformin could significantly alleviate increased FD4 flux caused by DSS, in a dose‐dependent manner. (Fig. 3A). After treated with metformin, the mRNA expression levels of TJ proteins ZO‐1 and occludin were significantly increased in colonic mucosa (Fig. 3B and C). Additionally, metformin treatment significantly improved TJ proteins expression (Fig. 3D and E). Furthermore, we collected colonic tissues from treated active UC patients with T2DM and assessed the expression levels of TJ proteins. We found that TJ proteins expression in patients with metformin treatment for T2DM were higher than those in patients with insulin treatment for T2DM (Fig. 3F and G), suggesting that metformin may participate in maintaining intestinal barrier function in UC patients.

### Metformin reduces commensal bacterial translocation in DSS‐induced colitis

Several studies have demonstrated that IBD patients and experimental colitis models have bacterial translocation, which can aggravate inflammation 26, 27. We extracted the bacterial DNA from MLNs, livers and spleens of the colitis models and performed quantitative PCR of bacterial 16S rDNA sequences. The average bacterial copies detected in the MLNs and livers, but not in the spleens, were significantly lower in metformin‐treated groups than that in DSS‐only group, although a significant difference was not observed between two metformin concentrations (Table 1).

**Table 1:** Bacterial translocation in colitis mouse models with or without metformin treatment

All eubacteria	Group			*P*
	DSS	DSS+100Met	DSS+500Met	
MLN	4.29 ± 0.62* ^,^ †	3.00 ± 0.30	3.26 ± 0.49	0.002
Liver	3.66 ± 0.17* ^,^ †	2.98 ± 0.16	3.02 ± 0.41	0.005
Spleen	2.92 ± 0.02	2.80 ± 0.05	2.77 ± 0.09	0.08

Bacterial translocation was determined by qRT‐PCR and expressed as log_10_ 16S rDNA gene copies. *P < 0.01, *versus* DSS+100Met; ^†^ *P* < 0.01, *versus* DSS+500Met.

### Metformin improves DSS‐induced barrier dysfunction *via* AMPKα1‐dependent inhibition of JNK activation

**Figure 4:**
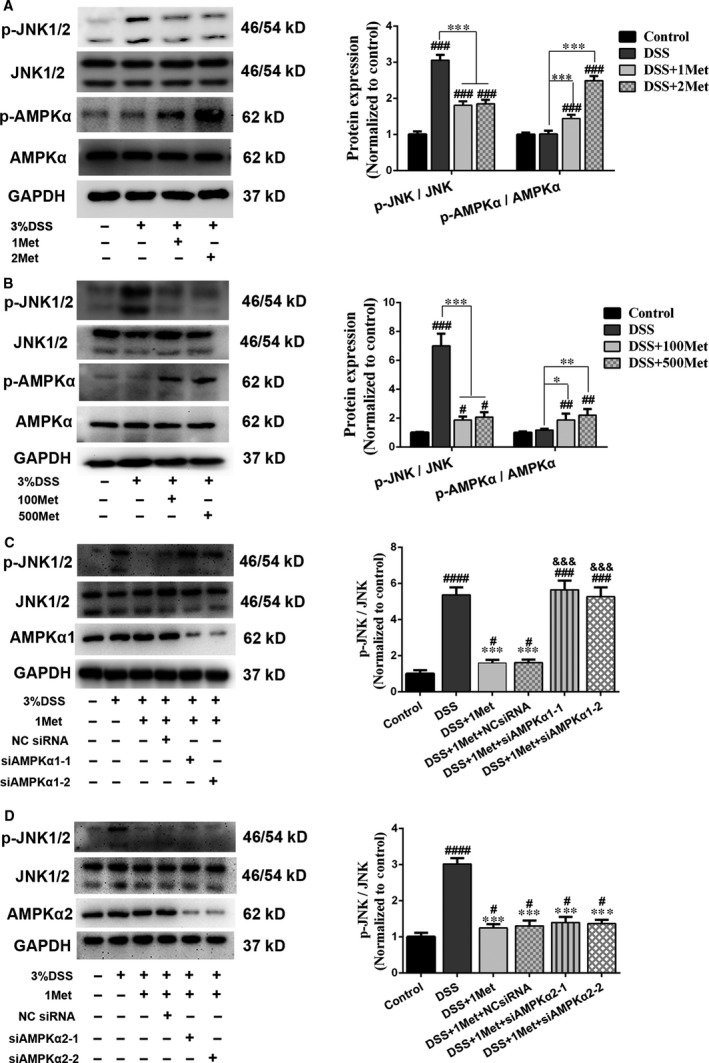
Metformin reduces DSS‐induced JNK activation *via* an AMPKα1‐dependent pathway. (**A**) After pre‐treatment with 1 mM / 2 mM metformin for 2 hrs, the Caco‐2 cell monolayers were costimulated with 3% DSS for 1 hrs. JNK1/2 and AMPKα phosphorylation levels were analysed. (**B**) The proteins extracted from colonic mucosa were immunoblotted for JNK1/2, p‐JNK1/2, AMPKα and p‐AMPKα. (**C–D**) Caco‐2 cell monolayers with siRNA‐mediated AMPKα1 and AMPKα2 knock‐down were exposed to DSS with or without metformin. JNK1/2 activation levels were determined by Western blotting. siAMPKα: AMPKα small interfering RNA, NC siRNA: negative control small interfering RNA. ^#^ *P* < 0.05; ^##^ *P* < 0.01; ^###^ *P* < 0.001; ^####^ *P* < 0.0001, *versus* control; *P < 0.05; **P < 0.01; ***P < 0.001, *versus* DSS; ^&&&^ *P* < 0.001, *versus* DSS+1Met.

JNK activation is involved in epithelial barrier dysfunction 28, 29. DSS can disrupt TJs by inducing JNK activation. This disruption can be attenuated by JNK inhibition or silencing 29. Therefore, we investigated whether metformin improved barrier function by inhibiting JNK activation. DSS‐induced JNK activation was significantly reduced by metformin treatment in Caco‐2 cell monolayers (Fig. 4A) or in intestinal epithelium (Fig. 4B). Moreover, AMPKα phosphorylation levels were higher after metformin administration (Fig. 4A and B). To determine whether metformin inhibited JNK activation through AMPKα‐dependent pathways, we used siRNA to silence AMPKα1 or AMPKα2 and found the inhibition of JNK activation by metformin was absent when AMPKα1, but not AMPKα2, was silenced (Fig. 4C and D), indicating that metformin suppresses DSS‐induced JNK activation *via* an AMPKα1‐dependent pathway.

**Figure 5:**
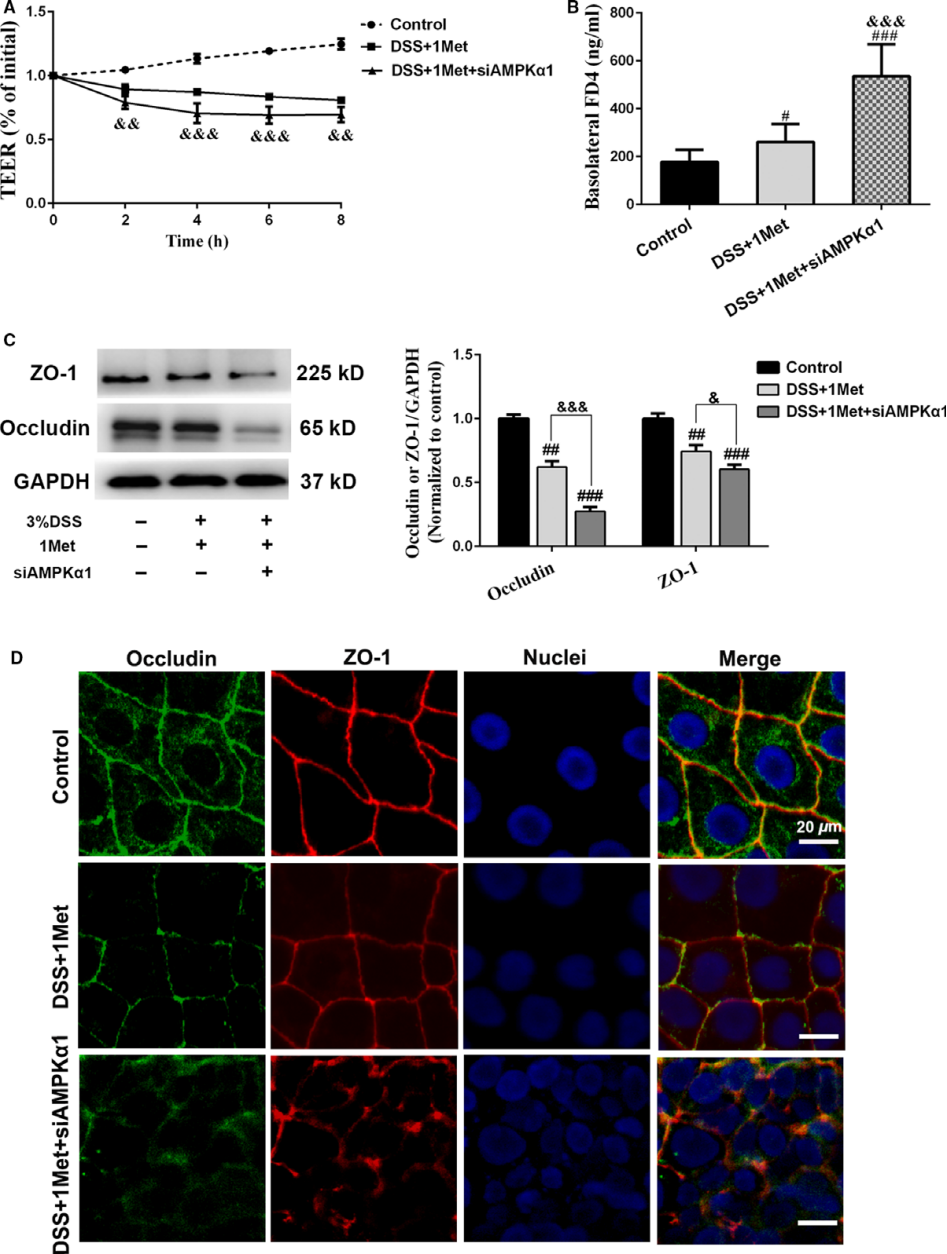
The impaired barrier‐protective effect of metformin in AMPKα1‐silenced cell monolayers with JNK activation. Caco‐2 cells were transfected with siAMPKα1 for 8 hrs, and then 5 × 10^5^ AMPKα1‐silenced cells were grown in 24‐well transwell chambers and cultured for 3 days until the TEER was above 300 Ω·cm^2^. Then 1 mM metformin pre‐treatment for 2 hrs, followed by coprocessing with 3% DSS for 8 hrs. (**A**) TEER values. (**B**) FD4 flux. (**C–D**) The protein levels of ZO‐1 and occludin were determined by immunoblot and immunofluorescence analysis. Values represent mean ± S.E.M. (*n* = 4). ^#^ *P* < 0.05; ^###^ *P* < 0.001, *versus* control; ^&&^ *P* < 0.01; ^&&&^ *P* < 0.001, *versus* DSS+1Met.

We also determined whether the barrier‐protective ability of metformin was impaired after AMPKα1 knock‐down. The data suggested that the DSS‐induced TEER decreases and FD4 flux increases were more significant in AMPKα1‐silenced cell monolayers after metformin treatment (Fig. 5A and B). We subsequently investigated the expression levels of ZO‐1 and occludin, and found that the protective effects of metformin were eliminated when AMPKα1 was knocked down (Fig. 5C). Moreover, immunofluorescence staining demonstrated that the continuity of abovementioned TJ proteins was disturbed more severely in AMPKα1‐silenced cell monolayers after DSS exposure (Fig. 5D). Taken together, these data confirmed that metformin improves DSS‐induced barrier dysfunction by inhibiting JNK activation *via* an AMPKα1‐dependent signalling pathway.

## Discussion

Intestinal epithelial barrier dysfunction is common in IBD patients and is considered an underlying pathophysiological component of the disease. Epithelial barrier integrity and function are mainly regulated by TJs 3. Metformin is now considered to have multiple beneficial effects on human body except as an antidiabetic drug. In the current study, we provided evidence that metformin can protect against intestinal barrier dysfunction through its effects on intestinal permeability and TJ proteins expression *in vitro* and *in vivo*. The mechanism underlying this protective effect may involve inhibiting JNK activation *via* an AMPKα1‐dependent signalling pathway.

The continuous physical barrier is composed of intestinal epithelial cells connected by TJs. TJs play an important role in the formation and maintenance of the intestinal epithelial barrier integrity. The disruption of TJ‐associated barrier integrity contributes to increased intestinal permeability and aggravating inflammation. Previous studies have demonstrated that mice with DSS‐induced colitis displayed intestinal barrier disruption and increased mucosal permeability 30, 31. We found that metformin protected against loss of the TJ proteins occludin and ZO‐1, decreases in TEER, FD4 hyperpermeability and bacterial translocation in Caco‐2 cell monolayers or in DSS‐induced colitis mice. This findings indicated that metformin could protect intestinal barrier function. The pathogenesis of bacterial translocation is believed to be related to intestinal barrier disruption and microbial dysbiosis in the gut 32. Intestinal hyperpermeability can increase bacterial penetration in IBD 33, 34. We found that metformin treatment significantly reduced bacteria translocation in the MLNs and livers. We think that the protective effects of metformin on barrier function may be contributed to inhibition of bacteria translocation.

There is little evidence that metformin has protective effects on barrier function in the present. The evidence collected by some researchers suggests that metformin can improve blood–brain barrier function 35, 36. A recent study reported that metformin treatment increased goblet cell differentiation and improved barrier function in IL‐10KO mice 37. Here, we showed that metformin protected against DSS‐induced intestinal barrier disruption by ameliorating TJs depletion and discontinuity. In clinical practice, metformin has not yet been used in the treatment of IBD. Some studies indicated that the incidence of T2DM is only 2–3% in UC as a concomitant disease 38, 39. We found that the expression of TJ proteins in active UC patients complicated with T2DM is higher in metformin treatment group than in insulin treatment group. According to our cells and animal experiments, it is very likely that the barrier‐protective effect of metformin is responsible for this phenomenon shown in UC patients with T2DM.

Studies have demonstrated that AMPK activation by metformin has anti‐inflammatory effect 13, 14. In our study, we showed that metformin could significantly prevent DSS‐induced colitis and inhibit production of the inflammatory factors IL‐6, TNF‐α and IL‐1β, providing evidence that metformin can attenuate the development of inflammation in colitis, a finding consistent with those of two other studies about the colitis remission induced by metformin treatment 17, 18. As the improvement of epithelial barrier function can prevent the development of intestinal inflammation 6, 8, we believe that this anti‐inflammatory effect of metformin is facilitated partly by maintaining intestinal barrier function.

The JNK family comprises three representative isoforms, JNK1, JNK2 and JNK3. JNK1 and JNK2 are expressed widely in many tissues 40. Studies have demonstrated that JNK is activated in the inflamed colonic epithelium of IBD patients 41, 42. Although the role of JNK activation in IBD is not clear, increasing amounts of evidence suggest that its activation plays a role in epithelial barrier dysfunction 28, 43. The epithelial junctions disrupted by JNK activation may be affected by multiple mechanisms, including impairment in adherens junction protein interactions, β‐catenin phosphorylation and altered TJ protein expression 44, 45. Inhibition of JNK activation can promote assembly of epithelial junctions and improve epithelial barrier function 28, 29, 44. Samak *et al*. 29 reported that DSS‐induced disruption of intestinal epithelial TJs was associated with JNK activation and the JNK inhibitor SP600125 could attenuate barrier dysfunction. In our study, we determined that metformin protects against DSS‐induced intestinal barrier disruption. We observed that metformin treatment could significantly alleviate DSS‐induced JNK activation and elevate AMPK phosphorylation. To determine the role of AMPK in metformin treatment‐induced JNK inhibition, we knocked down AMPKα1 or AMPKα2 and observed that the suppressive effects of metformin on JNK activation were abrogated after AMPKα1 knock‐down, as were its protective effects on intestinal barrier function. Some studies have reported that increased AMPK activation can suppress JNK signalling pathway phosphorylation levels 46, 47. In addition, some researchers suggested that AMPK activation can accelerate TJ assembly to enhance barrier functional integrity in MDCK cells with calcium depletion 48, 49. Taken together, these findings indicate that AMPKα1‐dependent inhibition of JNK signalling activation plays an important role in barrier‐ protective effect of metformin.

In conclusion, our current study indicated that metformin has protective effects on intestinal epithelial barrier function and attenuates intestinal inflammation in colitis. We noted that metformin ameliorates intestinal epithelial barrier dysfunction by inhibiting JNK, whose suppression is dependent on AMPKα1 activation. The detailed mechanism underlying this phenomenon still needs to be investigated. The findings presented in this study indicate that metformin can be served as a potential therapeutic medicine for IBD patients.

## Conflicts of interest

The authors declare no conflicts.

## Supporting information


**Table S1** Baseline demographic and clinical features of the study population.
**Table S2** The primer sequence used in this study.
**Table S3** The target Sequences of siRNA used in this study.

## References

[1] Turner JR . Intestinal mucosal barrier function in health and disease. Nat Rev Immunol. 2009; 9: 799–809. 19855405 10.1038/nri2653

[2] John LJ , Fromm M , Schulzke JD . Epithelial barriers in intestinal inflammation. Antioxid Redox Signal. 2011; 15: 1255–70. 21294654 10.1089/ars.2011.3892

[3] Ma TY , Iwamoto GK , Hoa NT , *et al* TNF‐alpha‐induced increase in intestinal epithelial tight junction permeability requires NF‐kappa B activation. Am J Physiol Gastrointest Liver Physiol. 2004; 286: G367–76. 14766535 10.1152/ajpgi.00173.2003

[4] Niessen CM . Tight junctions/adherens junctions: basic structure and function. J Invest Dermatol. 2007; 127: 2525–32. 17934504 10.1038/sj.jid.5700865

[5] Laukoetter MG , Nava P , Lee WY , *et al* JAM‐A regulates permeability and inflammation in the intestine *in vivo* . J Exp Med. 2007; 204: 3067–76. 18039951 10.1084/jem.20071416 PMC2150975

[6] Arrieta MC , Madsen K , Doyle J , *et al* Reducing small intestinal permeability attenuates colitis in the IL10 gene‐deficient mouse. Gut. 2009; 58: 41–8. 18829978 10.1136/gut.2008.150888 PMC2597688

[7] Edelblum KL , Turner JR . The tight junction in inflammatory disease: communication breakdown. Curr Opin Pharmacol. 2009; 9: 715–20. 19632896 10.1016/j.coph.2009.06.022 PMC2788114

[8] Arnott ID , Kingstone K , Ghosh S . Abnormal intestinal permeability predicts relapse in inactive Crohn disease. Scand J Gastroenterol. 2000; 35: 1163–9. 11145287 10.1080/003655200750056637

[9] Martin‐Montalvo A , Mercken EM , Mitchell SJ , *et al* Metformin improves healthspan and lifespan in mice. Nat Commun. 2013; 4: 2192–201. 23900241 10.1038/ncomms3192 PMC3736576

[10] Cabreiro F , Au C , Leung KY , *et al* Metformin retards aging in *C. elegans* by altering microbial folate and methionine metabolism. Cell. 2013; 153: 228–39. 23540700 10.1016/j.cell.2013.02.035 PMC3898468

[11] Shin NR , Lee JC , Lee HY , *et al* An increase in the Akkermansia spp. population induced by metformin treatment improves glucose homeostasis in diet‐induced obese mice. Gut. 2014; 63: 727–35. 23804561 10.1136/gutjnl-2012-303839

[12] Forslund K , Hildebrand F , Nielsen T , *et al* Disentangling type 2 diabetes and metformin treatment signatures in the human gut microbiota. Nature. 2015; 528: 262–6. 26633628 10.1038/nature15766 PMC4681099

[13] Hattori Y , Suzuki K , Hattori S , *et al* Metformin inhibits cytokine‐induced nuclear factor kappaB activation *via* AMP‐activated protein kinase activation in vascular endothelial cells. Hypertension. 2006; 47: 1183–8. 16636195 10.1161/01.HYP.0000221429.94591.72

[14] Sag D , Carling D , Stout RD , *et al* Adenosine 5′‐monophosphate‐activated protein kinase promotes macrophage polarization to an anti‐inflammatory functional phenotype. J Immunol. 2008; 181: 8633–41. 19050283 10.4049/jimmunol.181.12.8633 PMC2756051

[15] Son HJ , Lee J , Lee SY , *et al* Metformin attenuates experimental autoimmune arthritis through reciprocal regulation of Th17/Treg balance and osteoclastogenesis. Mediators Inflamm. 2014; 2014: 973986–99. 25214721 10.1155/2014/973986 PMC4158168

[16] Satapati S , Kucejova B , Duarte JA , *et al* Mitochondrial metabolism mediates oxidative stress and inflammation in fatty liver. J Clin Invest. 2015; 125: 4447–62. 26571396 10.1172/JCI82204 PMC4665800

[17] Koh SJ , Kim JM , Kim IK , *et al* Anti‐inflammatory mechanism of metformin and its effects in intestinal inflammation and colitis‐associated colon cancer. J Gastroenterol Hepatol. 2014; 29: 502–10. 24716225 10.1111/jgh.12435

[18] Lee SY , Lee SH , Yang EJ , *et al* Metformin Ameliorates Inflammatory Bowel Disease by Suppression of the STAT3 Signaling Pathway and Regulation of the between Th17/Treg Balance. PLoS One. 2015; 10: e0135858. 26360050 10.1371/journal.pone.0135858 PMC4567351

[19] Buzza MS , Netzel‐Arnett S , Shea‐Donohue T , *et al* Membrane‐anchored serine protease matriptase regulates epithelial barrier formation and permeability in the intestine. Proc Natl Acad Sci USA. 2010; 107: 4200–5. 20142489 10.1073/pnas.0903923107 PMC2840089

[20] Lewis JD , Chuai S , Nessel L , *et al* Use of the noninvasive components of the Mayo score to assess clinical response in ulcerative colitis. Inflamm Bowel Dis. 2008; 14: 1660–6. 18623174 10.1002/ibd.20520 PMC2597552

[21] Wang Z , Li R , Tan J , *et al* Syndecan‐1 acts in synergy with tight junction through Stat3 signaling to maintain intestinal mucosal barrier and prevent bacterial translocation. Inflamm Bowel Dis. 2015; 21: 1894–907. 25970544 10.1097/MIB.0000000000000421

[22] Dieleman LA , Palmen MJ , Akol H , *et al* Chronic experimental colitis induced by dextran sulphate sodium (DSS) is characterized by Th1 and Th2 cytokines. Clin Exp Immunol. 1998; 114: 385–91. 9844047 10.1046/j.1365-2249.1998.00728.x PMC1905133

[23] Zhang Y , Wang Z , Liu J , *et al* Cell surface‐anchored syndecan‐1 ameliorates intestinal inflammation and neutrophil transmigration in ulcerative colitis. J Cell Mol Med. 2017; 21: 13–25. 27558380 10.1111/jcmm.12934 PMC5192823

[24] Ma TY . Intestinal epithelial barrier dysfunction in Crohn's disease. Proc Soc Exp Biol Med. 1997; 214: 318–27. 9111522 10.3181/00379727-214-44099

[25] Zeissig S , Burgel N , Gunzel D , *et al* Changes in expression and distribution of claudin 2, 5 and 8 lead to discontinuous tight junctions and barrier dysfunction in active Crohn's disease. Gut. 2007; 56: 61–72. 16822808 10.1136/gut.2006.094375 PMC1856677

[26] O'Brien CL , Pavli P , Gordon DM , *et al* Detection of bacterial DNA in lymph nodes of Crohn's disease patients using high throughput sequencing. Gut. 2014; 63: 1596–606. 24429583 10.1136/gutjnl-2013-305320

[27] Peyrin‐Biroulet L , Gonzalez F , Dubuquoy L , *et al* Mesenteric fat as a source of C reactive protein and as a target for bacterial translocation in Crohn's disease. Gut. 2012; 61: 78–85. 21940721 10.1136/gutjnl-2011-300370 PMC3230831

[28] Naydenov NG , Hopkins AM , Ivanov AI . c‐Jun N‐terminal kinase mediates disassembly of apical junctions in model intestinal epithelia. Cell Cycle. 2009; 8: 2110–21. 19502798 10.4161/cc.8.13.8928

[29] Samak G , Chaudhry KK , Gangwar R , *et al* Calcium/Ask1/MKK7/JNK2/c‐Src signalling cascade mediates disruption of intestinal epithelial tight junctions by dextran sulfate sodium. Biochem J. 2015; 465: 503–15. 25377781 10.1042/BJ20140450 PMC4385020

[30] van Meeteren ME , van Bergeijk JD , van Dijk AP , *et al* Intestinal permeability and contractility in murine colitis. Mediators Inflamm. 1998; 7: 163–8. 9705603 10.1080/09629359891090 PMC1781840

[31] Poritz LS , Garver KI , Green C , *et al* Loss of the tight junction protein ZO‐1 in dextran sulfate sodium induced colitis. J Surg Res. 2007; 140: 12–9. 17418867 10.1016/j.jss.2006.07.050

[32] Clements WD , Parks R , Erwin P , *et al* Role of the gut in the pathophysiology of extrahepatic biliary obstruction. Gut. 1996; 39: 587–93. 8944570 10.1136/gut.39.4.587 PMC1383274

[33] Li P , Hotamisligil GS . Metabolism: host and microbes in a pickle. Nature. 2010; 464: 1287–8. 20428156 10.1038/4641287a

[34] Johansson ME , Gustafsson JK , Holmen‐Larsson J , *et al* Bacteria penetrate the normally impenetrable inner colon mucus layer in both murine colitis models and patients with ulcerative colitis. Gut. 2014; 63: 281–91. 23426893 10.1136/gutjnl-2012-303207 PMC3740207

[35] Takata F , Dohgu S , Matsumoto J , *et al* Metformin induces up‐regulation of blood‐brain barrier functions by activating AMP‐activated protein kinase in rat brain microvascular endothelial cells. Biochem Biophys Res Commun. 2013; 433: 586–90. 23523792 10.1016/j.bbrc.2013.03.036

[36] Liu Y , Tang G , Li Y , *et al* Metformin attenuates blood‐brain barrier disruption in mice following middle cerebral artery occlusion. J Neuroinflammation. 2014; 11: 177–89. 25315906 10.1186/s12974-014-0177-4 PMC4201919

[37] Xue Y , Zhang H , Sun X , *et al* Metformin Improves Ileal Epithelial Barrier Function in Interleukin‐10 Deficient Mice. PLoS One. 2016; 11: e0168670. 28002460 10.1371/journal.pone.0168670 PMC5176295

[38] Ha C , Magowan S , Accortt NA , *et al* Risk of arterial thrombotic events in inflammatory bowel disease. Am J Gastroenterol. 2009; 104: 1445–51. 19491858 10.1038/ajg.2009.81

[39] Dregan A , Charlton J , Chowienczyk P , *et al* Chronic inflammatory disorders and risk of type 2 diabetes mellitus, coronary heart disease, and stroke: a population‐based cohort study. Circulation. 2014; 130: 837–44. 24970784 10.1161/CIRCULATIONAHA.114.009990

[40] Bogoyevitch MA , Ngoei KR , Zhao TT , *et al* c‐Jun N‐terminal kinase (JNK) signaling: recent advances and challenges. Biochim Biophys Acta. 2010; 1804: 463–75. 19900593 10.1016/j.bbapap.2009.11.002

[41] Waetzig GH , Seegert D , Rosenstiel P , *et al* p38 mitogen‐activated protein kinase is activated and linked to TNF‐alpha signaling in inflammatory bowel disease. J Immunol. 2002; 168: 5342–51. 11994493 10.4049/jimmunol.168.10.5342

[42] Scaldaferri F , Sans M , Vetrano S , *et al* The role of MAPK in governing lymphocyte adhesion to and migration across the microvasculature in inflammatory bowel disease. Eur J Immunol. 2009; 39: 290–300. 19130554 10.1002/eji.200838316

[43] Samak G , Narayanan D , Jaggar JH , *et al* CaV1.3 channels and intracellular calcium mediate osmotic stress‐induced N‐terminal c‐Jun kinase activation and disruption of tight junctions in Caco‐2 CELL MONOLAYERS. J Biol Chem. 2011; 286: 30232–43. 21737448 10.1074/jbc.M111.240358 PMC3191062

[44] Lee MH , Koria P , Qu J , *et al* JNK phosphorylates beta‐catenin and regulates adherens junctions. FASEB J. 2009; 23: 3874–83. 19667122 10.1096/fj.08-117804 PMC2774999

[45] Carrozzino F , Pugnale P , Feraille E , *et al* Inhibition of basal p38 or JNK activity enhances epithelial barrier function through differential modulation of claudin expression. Am J Physiol Cell Physiol. 2009; 297: C775–87. 19605737 10.1152/ajpcell.00084.2009

[46] Filippov S , Pinkosky SL , Lister RJ , *et al* ETC‐1002 regulates immune response, leukocyte homing, and adipose tissue inflammation *via* LKB1‐dependent activation of macrophage AMPK. J Lipid Res. 2013; 54: 2095–108. 23709692 10.1194/jlr.M035212 PMC3708360

[47] Ma Y , Wang J , Gao J , *et al* Antithrombin up‐regulates AMP‐activated protein kinase signalling during myocardial ischaemia/reperfusion injury. Thromb Haemost. 2015; 113: 338–49. 25230600 10.1160/TH14-04-0360 PMC4308562

[48] Zheng B , Cantley LC . Regulation of epithelial tight junction assembly and disassembly by AMP‐activated protein kinase. Proc Natl Acad Sci USA. 2007; 104: 819–22. 17204563 10.1073/pnas.0610157104 PMC1783397

[49] Zhang L , Li J , Young LH , *et al* AMP‐activated protein kinase regulates the assembly of epithelial tight junctions. Proc Natl Acad Sci USA. 2006; 103: 17272–7. 17088526 10.1073/pnas.0608531103 PMC1859922

