# Supplementary material for: Metformin protects against intestinal barrier dysfunction via AMPKα1‐dependent inhibition of JNK signalling activation
Source: J Cell Mol Med. 2017 Nov 17;22(1):546–57. doi: 10.1111/jcmm.13342 (PMC5742676; doi:10.1111/jcmm.13342)
Supplement: Supplementary file 1 — Table S1 Baseline demographic and clinical features of the study population. Table S2 The primer sequence used in this study. Table S3 The target Sequences of siRNA used in this study. [file JCMM-22-546-s001.docx]

**Table S1 Baseline demographic and clinical features of the study population**

|  | UC with T2DM | | Controls |
| --- | --- | --- | --- |
|  | Metformin | Insulin |  |
| patients | 8 | 14 | 20 |
| Gender(male/female) | 5/3 | 10/4 | 12/8 |
| Median age in years  (IQR) | 48.5（42.75-55.5） | 46.5（40.75-50.75） | 37（32-41.5） |
| Median partial Mayo score  (IQR) | 5.5（2.5-6.75） | 7.5（4.25-9.25） |  |
| Active/Remission | 6/2 | 11/3 |  |
| Disease extent |  |  |  |
| Proctitis | 0 | 0 |  |
| Left-sided colitis | 4 | 6 |  |
| Extensive colitis | 4 | 8 |  |
| Medications |  |  |  |
| 5-ASA | 3 | 5 |  |
| Steroids | 3 | 6 |  |
| AZA | 2 | 3 |  |

ASA: aminosalicylic acid; AZA: azathioprine; IQR: interquartile range

**Table S2 The primer sequence used in this study**

| Primer name | Primer sequence (5′-3′) |
| --- | --- |
| IL-6 | Forward: TAGTCCTTCCTACCCCAATTTCC |
|  | Reverse: TTGGTCCTTAGCCACTCCTTC |
| TNF-α | Forward: CCTGTAGCCCACGTCGTAG |
|  | Reverse: GGGAGTAGACAAGGTACAACCC |
| IL-1β | Forward: CTCGTGCTGTCGGACCCAT |
|  | Reverse: CAGGCTTGTGCTCTGCTTGTGA |
| Zo-1 | Forward: GCCTCATCTCCAGTCCC- TTAC |
|  | Reverse: GCAATGGTGGTCCTTCACCT |
| occludin | Forward: GATGCAGGTC- TGCAGGAGTATAA |
|  | Reverse: ATCCTTAATTGGAGTGTTCAGCC |
| β-actin | Forward: AGAGGGAAATCGTGCGTGAC |
|  | Reverse: CAATAGTGATGACCTGGCCGT |

**Table S3 The target Sequences of siRNA used in this study**

| Gene name | Sequences |
| --- | --- |
| siAMPKα1-1 | Sense: CGGGAUCAGUUAGCAACUATT |
|  | Antisense: UAGUUGCUAACUGAUCCCGTT |
| siAMPKα1-2 | Sense: GAGGAGAGCUAUUUGAUUATT |
|  | Antisense: UAAUCAAAUAGCUCUCCUCTT |
| siAMPKα2-1 | Sense: CCACUCUCCUGAUGCAUAUTT |
|  | Antisense: AUAUGCAUCAGGAGAGUGGTT |
| siAMPKα2-2 | Sense: GGCUCUUUCAGCAGAUUCUTT |
| NC siRNA | Antisense: AGAAUCUGCUGAAAGAGCCTT  Sense: UUCUCCGAACGUGUCACGUTT  Antisense: ACGUGACACGUUCGGAGAATT |
